# Toward equitable digital health: an integrated framework addressing exclusion, ethics, and implementation across healthcare systems

**DOI:** 10.1186/s12939-026-02903-1

**Published:** 2026-05-29

**Authors:** Suresh Bangla, Anuva Kapoor, Girish Jeer

**Affiliations:** 1 Centre for Community Medicine, AIIMS, Delhi, India; 2 Community Medicine, AIIMS, Nagpur, India; 3 Department of Community Medicine, Kasturba Medical College Mangalore, Manipal Academy of Higher Education, Manipal, India

**Keywords:** Digital health equity, Health disparities, Digital divide, Artificial intelligence ethics, Algorithmic bias, Cybersecurity, Implementation science, Informed consent, Data governance, Health information technology

## Abstract

**Background:**

Digital health technologies are fundamentally reshaping healthcare delivery, access, and governance worldwide. While these innovations offer unprecedented opportunities to extend services, particularly in resource-constrained settings, early implementation experiences from large-scale platforms such as India’s Co-WIN vaccination system and the Ayushman Bharat Digital Mission (ABDM) reveal that poorly designed digitalization can entrench or even widen existing health inequities. Despite growing recognition of these challenges, there remains an urgent need for a coherent analytical framework to understand how patterns of digital exclusion, ethical challenges, and implementation barriers interact to shape digital health outcomes across diverse healthcare systems.

**Objectives:**

This narrative review aims to: (1) synthesize current evidence on patterns and mechanisms of digital health exclusion across socioeconomic, geographic, and demographic dimensions; (2) identify key ethical and governance challenges, including informed consent, data protection, algorithmic fairness, and cybersecurity, in contemporary digital health implementations; (3) analyze implementation experiences from India and comparable low- and middle-income country (LMIC) settings; and (4) propose an integrated equity framework to guide more inclusive digital health strategies that can inform policy, practice, and future research.

**Methods:**

We undertook a narrative review of peer-reviewed literature, policy documents, and grey literature published between 2015 and 2025 to examine digital health equity, ethical governance, and implementation challenges. Relevant sources were identified through iterative, purposive searches of PubMed/MEDLINE, Scopus, Web of Science, and the WHO Global Health Library, supplemented by targeted grey literature retrieval. These searches identified 312 records; following deduplication and eligibility screening, 22 peer-reviewed and policy sources were included in the synthesis. No AI-assisted screening tools were employed. Literature addressing patterns of digital exclusion, ethical or legal considerations, and real-world implementation experiences in clinical or public health settings was conceptually and thematically synthesized to identify recurrent themes, interconnections, and critical gaps in current digital health practice.

**Results:**

Analysis revealed that digital health interventions frequently mirror and amplify offline health inequities. Structural barriers related to income, gender, geography, disability, age, and digital literacy shape who owns devices, controls personal health data, and can meaningfully engage with digital health tools. Ethical challenges, including inadequate consent pathways, fragmented data protection regimes, persistent algorithmic bias, and escalating cybersecurity risks, remain pervasive across many national programs. Implementation experiences from India and similar contexts highlight fragmented governance structures, uneven state-level adoption, limited provider capacity, and significant risks associated with “digital-by-default” service delivery models that marginalize non-digital access options. Three critical implementation gaps emerged: (1) insufficient participatory design processes, (2) weak equity monitoring systems, and (3) limited accountability mechanisms for addressing exclusion or harm.

**Conclusions:**

Digital health will not inherently advance health equity; rather, equity must be intentionally designed, systematically governed, and continuously monitored throughout the implementation lifecycle. We propose an integrated Digital Health Equity Framework that explicitly links patterns of exclusion with ethical safeguards and implementation strategies. Core components include: participatory co-design with marginalized communities, privacy-by-design and safety-by-design architectures, multi-channel service delivery that preserves non-digital pathways, routine disaggregated equity monitoring, and transparent algorithmic governance with redress mechanisms. Application of this framework can help policymakers, health system leaders, and technology developers ensure that national digital health programs, in India and globally, close rather than widen equity gaps, ultimately advancing universal health coverage through inclusive digital transformation.

**Supplementary Information:**

The online version contains supplementary material available at 10.1186/s12939-026-02903-1.

## Introduction

Healthcare systems worldwide are undergoing a profound digital transition that is fundamentally reshaping how care is accessed, delivered, monitored, and governed [1]. This transformation has elevated the importance of what are increasingly termed the *digital determinants of health*, the constellation of technological access, digital literacy, data governance structures, and algorithmic performance characteristics that now interact dynamically with traditional social determinants to influence health outcomes [2, 3].

Yet, this digital transformation has surfaced a central paradox. The same technologies that promise to democratize healthcare access, strengthen continuity of care, reduce geographic barriers, and improve population health surveillance can simultaneously create new vectors of exclusion and exacerbate existing health inequities [4]. These inequities emerge from multiple intersecting sources: gaps in connectivity infrastructure and device ownership, variable digital literacy across populations, inadequate accessibility features for persons with disabilities, opaque data practices that undermine informed consent, and algorithmic biases embedded within AI-driven clinical decision support systems [3, 5].

Early evidence from large-scale digital health implementations illustrates these tensions vividly. India’s Co-WIN platform, deployed to coordinate COVID-19 vaccination at unprecedented scale, achieved remarkable reach, yet also revealed systematic exclusion of elderly populations, rural residents, and individuals lacking smartphones or digital literacy [4]. Similarly, the Ayushman Bharat Digital Mission (ABDM), India’s ambitious national digital health infrastructure, has encountered significant implementation challenges including fragmented governance, uneven state-level adoption, limited provider capacity, and concerns about privacy protection and data security [4].

These implementation experiences underscore a critical insight: digital health equity is not a technical problem alone, but rather a complex sociotechnical challenge requiring integrated attention to patterns of exclusion, ethical governance, and implementation strategy [6, 7]. Existing frameworks have tended to address these dimensions in isolation, focusing either on access barriers, or ethical principles, or implementation factors, without adequately capturing their dynamic interactions [8].

This narrative review addresses this gap by synthesizing current evidence across these three critical domains and proposing an integrated Digital Health Equity Framework. Our analysis is grounded in implementation experiences from India and comparable LMIC settings, where resource constraints, infrastructure limitations, and diverse population needs make equity considerations particularly acute. However, the framework’s principles have broader applicability to high-income settings confronting their own digital health equity challenges.

The paper proceeds as follows: Section "Methods" describes our review methodology; Section "Narrative synthesis findings: patterns of digital health exclusion" examines patterns and mechanisms of digital health exclusion; Section "Ethical and security challenges in digital health" analyzes ethical and security challenges; Section "Implementation experiences and barriers" reviews implementation experiences and barriers; Section "An integrated digital health equity framework" presents our integrated equity framework; and Section "Discussion" discusses implications for policy, practice, and research.

## Methods

### Literature identification

Relevant literature was identified through iterative, non-exhaustive searches of major biomedical and multidisciplinary databases, including PubMed/MEDLINE, Scopus, Web of Science, and the WHO Global Health Library. Searches were conducted for materials published between 2015 and 2025 to capture the period of rapid expansion of national digital health platforms, particularly following the COVID-19 pandemic.

Search terms were purposively selected to reflect three intersecting domains central to the review objectives:

- Digital health technologies (e.g., digital health, eHealth, mHealth, telemedicine, health information systems, artificial intelligence in healthcare).
- Equity and access dimensions (e.g., health equity, digital divide, health disparities, marginalized populations).
- Ethics and implementation considerations (e.g., informed consent, data privacy, algorithmic bias, cybersecurity, implementation science).

In addition to database searches, key policy documents, program reports, and grey literature were identified through targeted searches of institutional websites and by screening reference lists of highly cited and conceptually influential publications.

### Eligibility and selection

Sources were considered eligible if they addressed at least one of the following: inequities in access to or use of digital health technologies; ethical, legal, or governance challenges in digital health systems; implementation experiences of digital health interventions in clinical or public health settings; or conceptual or policy frameworks relevant to digital health equity. Table 1 details the full inclusion and exclusion criteria applied.

**Table 1:** Inclusion and exclusion criteria applied during literature screening

Criterion	Inclusion	Exclusion
Study types	Empirical quantitative and qualitative studies, systematic and scoping reviews, policy analyses, national program evaluations, conceptual frameworks	Opinion pieces without substantive analytical content; studies focusing solely on technical performance without equity relevance
Phenomena of interest	Digital health equity and exclusion; ethical, legal, or governance challenges; real-world implementation experiences; conceptual or policy frameworks for digital health equity	Technical performance evaluations without equity relevance; hospital IT infrastructure studies unrelated to patient-facing outcomes
Context	Any healthcare setting; emphasis on LMICs and India; any country	Non-health digital equity studies without linkage to health outcomes
Publication period	2015–2025	Before 2015; non-English publications
Quality	No formal quality scoring (narrative design); greater interpretive weight given to recent systematic reviews, large-scale implementation studies, and authoritative policy sources	Predatory journal publications; sources without identifiable institutional affiliation; full text not retrievable after two retrieval attempts

Database searches across the four sources yielded 312 records. Following removal of 65 duplicates, 247 records were screened by title and abstract; 179 were excluded as clearly out of scope. Sixty-eight records proceeded to full-text review; 47 were excluded (insufficient equity focus, *n* = 18; technical performance only, *n* = 14; opinion without analysis, *n* = 9; full text not retrievable, *n* = 6). Twenty-two peer-reviewed publications and policy documents met all eligibility criteria and were included in the final synthesis. A complete literature flow diagram is provided in Supplementary File 1. No AI-assisted screening tools were employed at any stage; all title–abstract and full-text screening was conducted manually by the authors.

### Synthesis approach

We employed thematic synthesis methods to identify convergent patterns across studies. Initial coding was inductive, allowing themes to emerge from the data. Through iterative analysis, we organized findings into three primary domains: (1) patterns of digital health exclusion, (2) ethical and security challenges, and (3) implementation experiences and barriers. Cross-cutting themes were then identified to inform the integrated framework presented in Section "An integrated digital health equity framework".

## Narrative synthesis findings: patterns of digital health exclusion

Our analysis identified multiple intersecting dimensions through which digital health interventions can perpetuate or exacerbate health inequities. We organize these patterns using an adapted exclusion typology that distinguishes between structural barriers (rooted in socioeconomic and infrastructure factors) and personal/social barriers (related to individual characteristics and social contexts) [3, 9]. Table 2 provides a comprehensive overview of eight types of digital health exclusion.

**Table 2:** Types of digital health exclusion, definitions, examples, and affected populations

Type of exclusion	Definition	Examples in healthcare	Affected populations
1. Access Exclusion	Lack of physical access to digital devices, internet connectivity, or digital health platforms	No internet connection, lack of smartphone/computer, poor broadband infrastructure in rural areas	Rural populations, low-income households, elderly individuals
2. Skills Exclusion	Insufficient digital literacy or technical skills to effectively use health technologies	Cannot navigate health apps, difficulty using telehealth platforms, unable to understand digital interfaces	Older adults, individuals with low education levels, people with cognitive impairments
3. Usage Exclusion	Inability to engage meaningfully with digital health tools despite having access and skills	Has access and skills but doesn’t use telehealth due to preference for face-to-face care	Individuals preferring traditional care, those with negative past experiences
4. Outcome Exclusion	Failure to derive health benefits from digital technologies due to various barriers	Uses digital health tools but doesn’t achieve improved health outcomes due to inappropriate design	Marginalized communities, individuals with complex health needs, culturally diverse populations
5. Motivation Exclusion	Lack of interest, confidence, or perceived need to use digital health services	Prefers traditional healthcare methods, lacks trust in digital technologies, fears privacy breaches	Elderly patients, individuals with health anxiety, privacy-conscious populations
6. Physical Exclusion	Physical disabilities or limitations that prevent effective use of digital health technologies	Visual impairments affecting screen reading, motor difficulties with touch screens, hearing loss affecting video calls	People with disabilities, individuals with visual/hearing/motor impairments
7. Economic Exclusion	Financial barriers preventing access to devices, internet services, or digital health platforms	Cannot afford internet plans, smartphones, or subscription-based health apps	Low-income families, unemployed individuals, people in developing regions
8. Cultural/Social Exclusion	Language barriers, cultural incompatibility, or social exclusion from digital health services	Non-English speaking patients, cultural preference for in-person care, stigma around mental health apps	Ethnic minorities, non-English speakers, socially isolated individuals

### Structural barriers to digital health access

#### Socioeconomic dimensions of the digital divide

The digital divide, disparities in access to information and communication technologies, remains a fundamental driver of digital health inequity [2]. Income stratification shapes multiple dimensions of digital access: device ownership (smartphones, tablets, computers), quality of internet connectivity (broadband vs. mobile data), and ability to afford data plans for health-related applications [3].

Evidence from LMIC settings reveals stark gradients. In India, smartphone penetration varies from over 75% in urban areas to less than 30% in rural regions, with even wider gaps among economically disadvantaged populations [4]. These disparities directly constrain access to digital health services: individuals without smartphones cannot use mobile health applications, participate in telemedicine consultations, or access digital health records, effectively creating a two-tier healthcare system stratified by technological access [9].

Even in high-income settings, socioeconomic stratification shapes digital health engagement. Studies from the United States demonstrate that lower-income patients are significantly less likely to use patient portals, engage with remote monitoring programs, or participate in telehealth visits, not due to lack of interest, but due to inadequate devices, unreliable internet access, or inability to afford data costs [2, 7]. The WHO Global Strategy on Digital Health 2020–2025 recognizes equitable access as a foundational precondition for advancing universal health coverage through digital transformation, cautioning that poorly governed digitalization can widen rather than close health divides [10].

#### Rural-Urban geographic disparities

Geographic location intersects with socioeconomic status to create compounding barriers. Rural and remote populations face dual challenges: limited telecommunications infrastructure (poor network coverage, slow connection speeds) and lower availability of digitally-enabled healthcare services [3]. Even when digital health programs are theoretically available nationwide, implementation often prioritizes urban centers where infrastructure is more robust and provider capacity higher [4].

India’s experience with the ABDM illustrates these dynamics. While the platform aims to provide universal digital health coverage, adoption has been heavily concentrated in urban tertiary hospitals, with minimal penetration in rural primary health centers where infrastructure limitations, provider digital literacy gaps, and patient access barriers converge [4]. This pattern risks creating a paradox where digital health, ostensibly intended to reduce geographic barriers, instead widens the rural-urban health divide.

### Personal and social barriers to digital health engagement

#### Gender dimensions of digital health exclusion

Gender operates as a critical axis of digital health exclusion through multiple mechanisms. In many LMIC contexts, women have substantially lower rates of smartphone ownership and internet access compared to men, reflecting broader patterns of economic inequality and social norms around technology use [11]. Even within households with shared devices, women often have limited or controlled access, mediated by male family members [9].

Beyond access, gender shapes the design and implementation of digital health services in ways that can perpetuate exclusion. Many digital health applications lack features addressing women’s specific health needs (reproductive health, maternal care, gender-based violence resources) or fail to account for privacy and safety concerns particularly salient for women [11]. Algorithmic bias in AI-driven clinical tools has also been documented, with some diagnostic or risk prediction algorithms performing less accurately for women due to training data imbalances [5].

#### Age-related digital divides

Age represents another dimension of systematic exclusion. Older adults face multiple barriers: lower digital literacy, physical limitations (vision, hearing, dexterity) that impair device use, cognitive factors affecting interface navigation, and often lower confidence or trust in digital technologies [9]. These barriers are particularly concerning given that older populations often have the greatest healthcare needs and could benefit substantially from digital health services.

India’s Co-WIN experience provides a stark illustration. Despite older adults being priority populations for COVID-19 vaccination, the platform’s smartphone-dependent registration and appointment system systematically excluded elderly individuals lacking devices, digital literacy, or family support for navigation [4]. Similar patterns have been documented globally, with telemedicine and patient portal adoption consistently lower among older adults [2].

#### Disability and accessibility gaps

Persons with disabilities face particularly severe digital health exclusion due to inadequate accessibility features in most digital health platforms [3]. Visual impairments, hearing loss, motor limitations, and cognitive disabilities each require specific design accommodations, screen reader compatibility, captioning, simplified navigation, adjustable text size, that are frequently absent from health applications and telemedicine platforms [9].

This exclusion is especially problematic because persons with disabilities often have complex healthcare needs requiring frequent monitoring and coordination. Digital health tools could theoretically reduce barriers to care access, yet when designed without accessibility considerations, they instead create new barriers that compound existing ones [3].

#### Digital literacy and skills exclusion

Beyond physical access to devices and connectivity, effective engagement with digital health requires substantial digital literacy, the ability to navigate interfaces, input information accurately, interpret results, and troubleshoot technical problems [9]. Digital literacy is not uniformly distributed; it correlates strongly with education level, age, socioeconomic status, and prior technology exposure [2].

Many digital health platforms assume high baseline digital competency, with complex registration processes, multi-step navigation, and limited guidance or support [3]. When users encounter difficulties, help resources are often inadequate, available only online (creating a circular barrier), or require literacy in dominant languages that may not be users’ primary language [9].

Provider digital literacy is equally critical yet often overlooked. Healthcare workers, particularly in resource-constrained settings, may lack training in digital health systems, leading to incomplete implementation, workarounds that undermine data quality, or resistance to adoption [4]. Successful digital health implementation requires sustained investment in provider training and ongoing technical support [7].

## Ethical and security challenges in digital health

Beyond access and exclusion, digital health raises profound ethical and security challenges that disproportionately affect vulnerable populations. We examine three critical domains: informed consent, algorithmic bias and AI ethics, and cybersecurity risks.

### Informed consent in digital health systems

#### The challenge of meaningful consent

Traditional models of informed consent, developed for discrete clinical encounters and research studies, are increasingly inadequate for digital health contexts characterized by continuous data collection, complex data sharing networks, and evolving uses of health information [12]. Digital health platforms typically present users with lengthy terms of service and privacy policies written in legal language, displayed on small screens, and requiring immediate acceptance to access services [12].

Research on digital consent demonstrates that users rarely read these documents, poorly understand the scope of data collection or sharing, and feel they have no meaningful choice but to accept given the necessity of accessing healthcare [12]. This creates what has been termed “consent theater” a term coined by Richards and Hartzog [12]- a procedural compliance with consent requirements that fails to achieve the ethical goal of informed, voluntary authorization.

#### Vulnerability and consent capacity

Consent challenges are particularly acute for vulnerable populations. Individuals with limited literacy, cognitive impairments, or limited digital experience may struggle to understand what they are consenting to [13]. Language barriers compound these difficulties when consent materials are available only in dominant languages [14].

Moreover, the power asymmetry inherent in healthcare relationships, where patients may fear that refusing consent will compromise their care, is amplified in digital contexts where consent is often required to access any services [13]. This is especially problematic in “digital-by-default” implementations where non-digital alternatives are eliminated or significantly degraded [4].

#### Dynamic consent models

Emerging “dynamic consent” approaches attempt to address these limitations by enabling ongoing, granular control over data use through interactive digital interfaces [15]. Rather than one-time acceptance of all terms, dynamic consent allows users to specify preferences for different types of data sharing, receive notifications of new uses, and modify permissions over time [15]. Research indicates that social annotation and peer cues within digital environments can meaningfully influence how users engage with consent interfaces, including their level of comprehension and the choices they make [16].

While promising, dynamic consent faces implementation challenges: it requires sophisticated technical infrastructure, may overwhelm users with frequent decisions, and has not been widely adopted in routine clinical care [15]. Moreover, it does not resolve fundamental power imbalances when essential services are conditioned on data sharing.

### Algorithmic bias and AI ethics

#### Sources and manifestations of algorithmic bias

Artificial intelligence and machine learning are increasingly embedded in digital health, for risk prediction, diagnostic support, treatment recommendations, and resource allocation [5]. However, numerous studies document significant biases in these algorithms, typically arising from three sources: biased training data, inappropriate feature selection, and biased optimization objectives [17, 18].

Training data bias occurs when datasets used to develop algorithms underrepresent certain populations or reflect historical patterns of healthcare inequality [5]. For example, if an algorithm is trained primarily on data from urban tertiary hospitals, it may perform poorly for rural or primary care populations whose clinical presentations and resource contexts differ [17].

Feature selection bias arises when algorithms rely on variables that serve as proxies for race, socioeconomic status, or other protected characteristics, thereby perpetuating discriminatory patterns [18]. A widely-cited example is the use of healthcare costs as a proxy for health needs in allocation algorithms, since marginalized populations often receive less care due to access barriers, they appear “healthier” to cost-based algorithms and are deprioritized for interventions [17].

#### Gender and racial bias in AI health systems

Gender bias in medical AI has been extensively documented. Many algorithms perform less accurately for women, particularly for conditions that present differently by sex or for which training data has been male-dominated [11]. Diagnostic algorithms for cardiovascular disease, for instance, have shown lower sensitivity for women due to training on predominantly male datasets and failure to account for sex-specific symptom patterns [11].

Racial and ethnic bias is equally pervasive. Studies demonstrate that AI diagnostic tools for dermatology perform poorly on darker skin tones due to training data skewed toward lighter skin [5]. Pulse oximetry algorithms, critical for COVID-19 management, have been shown to overestimate oxygen saturation in Black patients, potentially delaying necessary interventions [17].

#### Accountability and transparency deficits

A fundamental challenge is the lack of transparency in many AI health systems. Proprietary algorithms are often treated as “black boxes,” with developers unwilling to disclose training data, model architecture, or validation results [18]. This opacity prevents independent auditing, limits accountability when algorithms cause harm, and undermines trust, particularly among communities with historical experiences of medical discrimination [17].

Moreover, even when bias is identified, accountability mechanisms are often absent. Who is responsible when an algorithm contributes to misdiagnosis or inappropriate treatment, the developer, the healthcare organization, the clinician, or the algorithm itself? Current legal and regulatory frameworks provide limited clarity [5].

### Cybersecurity and privacy risks

#### Escalating cyber threats to health systems

Healthcare has become a prime target for cyberattacks due to the high value of health data, the critical nature of clinical systems, and often-inadequate security infrastructure [19]. Ransomware attacks on hospitals and health systems have escalated dramatically, with potentially life-threatening consequences when clinical systems are disabled [20].

Digital health platforms create expanded attack surfaces, more devices, more data flows, more third-party vendors, each representing potential vulnerabilities [21]. Mobile health applications, in particular, often have weak security controls, including inadequate encryption, insecure data storage, and poor authentication mechanisms [19].

#### Data breaches and privacy violations

Healthcare data breaches are alarmingly common. Analysis of reported incidents reveals that millions of patient records are compromised annually through hacking, unauthorized access, or inadvertent disclosure [22]. Breaches disproportionately affect vulnerable populations who may face greater harms, including discrimination, stigmatization, or financial fraud, from exposure of sensitive health information [22].

Privacy risks extend beyond malicious attacks to include inappropriate data sharing by healthcare organizations and commercial entities. Many digital health applications share user data with third-party advertisers, data brokers, or research organizations without adequate consent or transparency [12]. For marginalized communities with justified mistrust of institutions, these practices can deter engagement with digital health services altogether [2].

#### Regulatory gaps and enforcement challenges

Data protection regulations vary widely across jurisdictions, creating gaps that undermine privacy protection in cross-border digital health services [22]. Even where strong regulations exist (e.g., GDPR in Europe, HIPAA in the United States), enforcement is often inadequate, particularly for smaller digital health vendors or applications that fall outside traditional healthcare regulatory frameworks [21].

In many LMIC settings, data protection laws are absent or weakly enforced, leaving users with minimal legal recourse when privacy is violated [4]. This regulatory fragmentation is particularly problematic for national digital health infrastructures that aim to integrate data across multiple systems and jurisdictions [4].

## Implementation experiences and barriers

Moving from ethical principles to operational reality requires careful attention to implementation science, understanding how digital health interventions are translated into practice across diverse contexts [7]. We examine implementation experiences from India and comparable settings, identifying critical barriers and success factors.

### India’s digital health Journey: ABDM and Co-WIN

#### Ayushman Bharat Digital Mission (ABDM)

Launched in 2021, ABDM represents India’s ambitious vision for a unified national digital health infrastructure, encompassing unique health identifiers, digital health records, interoperability standards, and a registry of healthcare providers and facilities [4, 23]. The mission aims to enable seamless data exchange, improve care coordination, and support evidence-based policymaking.

However, implementation has encountered significant challenges:

##### Fragmented governance

Responsibility for digital health spans multiple ministries and agencies with overlapping mandates and limited coordination, creating confusion about roles, slowing decision-making, and impeding integration efforts [4, 23].

##### Uneven state adoption

India’s federal structure requires state-level implementation, but capacity, resources, and political commitment vary widely. Some states have made substantial progress while others have minimal ABDM presence, creating a patchwork of digital health maturity [4].

##### Provider capacity gaps

Many healthcare facilities, particularly primary health centers serving rural populations, lack adequate infrastructure (reliable electricity, internet connectivity, computers), trained personnel, and technical support to implement ABDM effectively [4].

##### Privacy and security concerns

Despite a data protection framework, concerns persist about consent mechanisms, data security, potential for surveillance, and lack of transparency about how health data is used or shared [4, 23].

##### Limited community engagement

Implementation has been largely top-down, with minimal involvement of patient communities, civil society organizations, or frontline health workers in design and governance decisions [4].

#### Co-WIN vaccination platform

Co-WIN was rapidly deployed in 2021 to coordinate India’s COVID-19 vaccination campaign, ultimately facilitating over 2 billion vaccine doses [4, 23]. While achieving impressive scale, the platform revealed critical equity gaps:

##### Digital-by-default exclusion

Initial implementation required smartphone-based registration and appointment booking, systematically excluding elderly populations, rural residents, and individuals without digital access or literacy [4].

##### Language and literacy barriers

The platform initially launched in English and Hindi only, excluding speakers of India’s numerous other languages. Even after multilingual expansion, navigation required literacy and digital competency [4].

##### Age verification challenges

Older adults faced particular difficulties with identity verification requirements, as many lacked the specific documents required or needed family assistance to navigate the system [4].

##### Adaptation and mitigation

Recognizing these barriers, authorities eventually implemented walk-in vaccination sites, offline registration options, and community support programs. However, these adaptations came after substantial exclusion had already occurred, illustrating the cost of not prioritizing equity from the outset [4, 23].

### Implementation lessons from comparable settings

#### Multi-channel service delivery

Evidence from diverse settings demonstrates that maintaining parallel non-digital service channels is essential for equity [7]. “Digital-by-default” approaches that eliminate or significantly degrade non-digital options systematically exclude populations facing access or literacy barriers [3].

Successful implementations preserve choice, allowing individuals to access services through their preferred modality, digital, in-person, telephone, or assisted digital [7]. This requires sustained investment in multiple channels rather than viewing digital as a cost-saving replacement for human service provision [9].

#### Participatory design and community engagement

Digital health interventions designed without meaningful input from intended users, particularly marginalized communities, frequently fail to meet needs, encounter resistance, or perpetuate exclusion [7]. Participatory design approaches that engage patients, community health workers, and advocacy organizations throughout the development process produce more acceptable, usable, and equitable solutions [6].

Effective participation requires more than token consultation. It demands genuine power-sharing, adequate time and resources for engagement, compensation for participants’ expertise, and demonstrable incorporation of community input into design decisions [7].

#### Provider training and support

Healthcare workforce capacity is consistently identified as a critical implementation barrier [4]. Providers require not only technical training in system use but also support for workflow integration, ongoing technical assistance when problems arise, and involvement in system refinement based on their frontline experience [7].

Training must be tailored to diverse provider contexts and capacities. Community health workers in rural areas have different needs than specialists in tertiary hospitals; one-size-fits-all training approaches are typically ineffective [4].

#### Infrastructure investment

Digital health cannot succeed without foundational infrastructure: reliable electricity, internet connectivity, hardware, and technical support [4]. In many LMIC settings, infrastructure limitations represent the primary implementation barrier, yet they are often overlooked in favor of software development and policy formulation [4].

Infrastructure investment must extend beyond healthcare facilities to communities themselves. If patients lack connectivity or devices at home, the benefits of digital health records, remote monitoring, or telemedicine cannot be realized [3].

### Critical implementation gaps

Synthesis of implementation evidence reveals three pervasive gaps:

**Gap 1: Insufficient Equity Monitoring**: Most digital health implementations lack systematic monitoring of equity outcomes, who is using services, who is excluded, whether disparities are widening or narrowing [6]. Without routine disaggregated data collection and analysis, inequities remain invisible and unaddressed [2].

**Gap 2: Weak Accountability Mechanisms**: When digital health systems cause harm, through exclusion, privacy breaches, algorithmic bias, or other failures, accountability pathways are often absent or ineffective [18]. Users need accessible mechanisms to report problems, seek redress, and influence system improvement [5].

**Gap 3: Limited Sustainability Planning**: Many digital health initiatives are project-based, with inadequate planning for long-term sustainability of infrastructure, training, technical support, and system maintenance [4]. When projects end and external funding ceases, systems often deteriorate or collapse [4].

## An integrated digital health equity framework

**Fig. 1:**
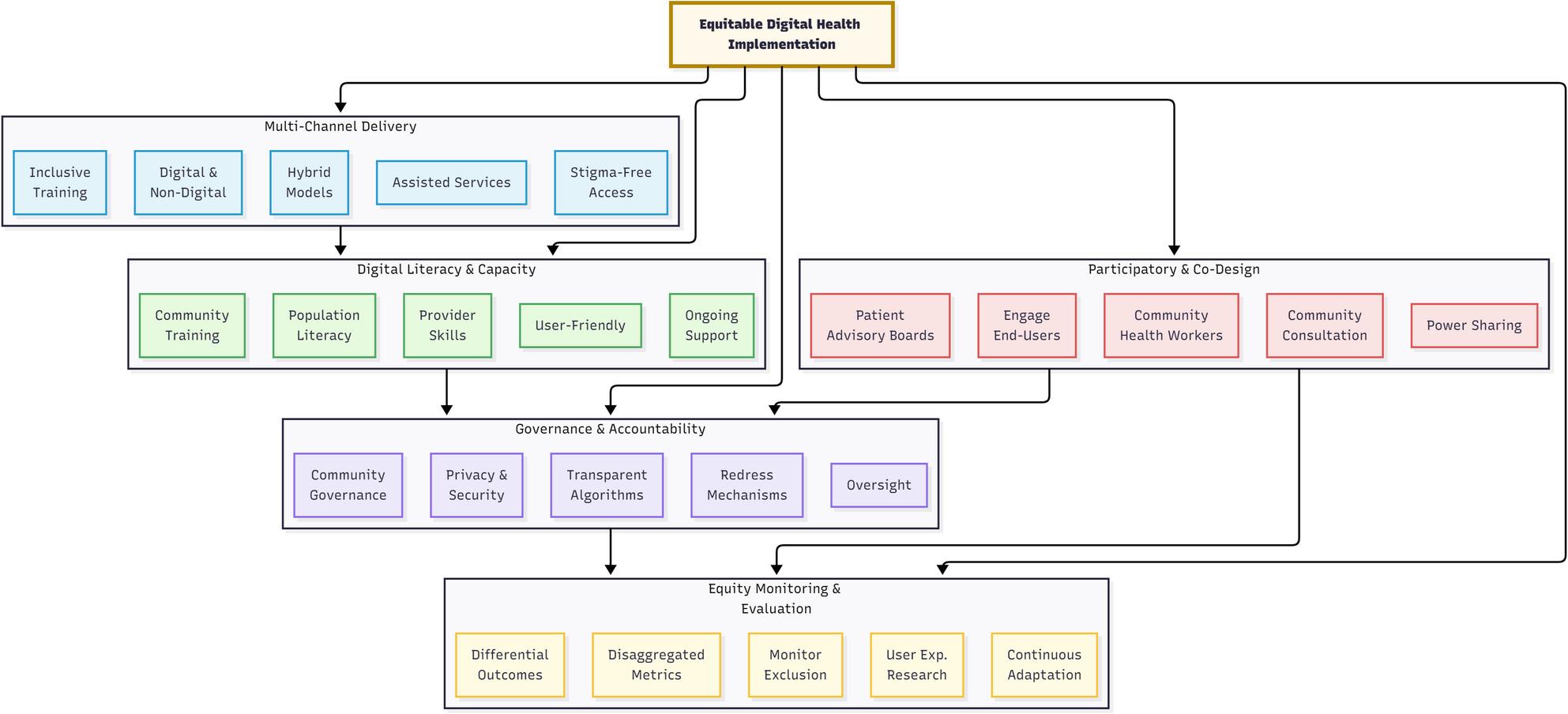
Integrated framework for equitable digital health implementation. This implementation framework translates the exclusion patterns and ethical challenges identified in Fig. 2 into actionable strategies organized across five interconnected domains. At the apex, Equitable Digital Health Implementation serves as the overarching goal. The framework flows through five key implementation domains: (1) Multi-Channel Delivery (shown in blue boxes) ensures that digital services complement rather than replace non-digital options, including inclusive training, hybrid models, assisted services, and stigma-free access; (2) Digital Literacy & Capacity (shown in green boxes) emphasizes building human capacity through community training, population literacy assessment, provider skills development, user-friendly design, and ongoing support; (3) Participatory & Co-Design (shown in pink boxes) centers the voices of affected communities through patient advisory boards, end-user engagement, community health worker involvement, community consultation, and genuine power sharing; (4) Governance & Accountability (shown in purple boxes) establishes transparent oversight through community governance structures, privacy and security protections, transparent algorithms, redress mechanisms, and independent oversight; and (5) Equity Monitoring & Evaluation (shown in yellow boxes) ensures continuous learning through differential outcome tracking, disaggregated metrics, exclusion monitoring, user experience research, and continuous adaptation. These five domains are mutually reinforcing, effective implementation requires attention to all domains simultaneously. The framework emphasizes that digital health equity is not a technical problem alone but a sociotechnical challenge requiring integrated attention to access, design, governance, and continuous learning

Building on the evidence synthesized above, we propose an integrated framework to guide equity-focused digital health implementation (Fig. 1). This framework explicitly links patterns of exclusion with implementation strategies and ethical safeguards, providing actionable guidance for policymakers, health system leaders, technology developers, and researchers.

While existing frameworks have substantially advanced the field, each addresses specific dimensions in relative isolation. Richardson et al. [2] mapped five domains centered on infrastructure access and digital literacy; Hatef et al. [6] developed a consensus-based framework emphasizing health system readiness; Kim and Backonja [8] conducted a scoping review identifying key conceptual components but generating a synthesis of existing concepts rather than a prescriptive model; and Groom et al. [7] constructed an implementation research model bridging equity and implementation science, focused primarily on the research process. None simultaneously integrates (i) a structured typology of digital exclusion mechanisms, (ii) explicit ethical governance requirements encompassing algorithmic accountability, consent architecture, and cybersecurity, and (iii) operational implementation strategy guidance, within a single model applicable to LMIC health system contexts. The framework proposed here addresses this gap through three specific contributions: first, tripartite integration of exclusion, ethics, and implementation within one actionable structure; second, grounding in LMIC implementation evidence including India’s ABDM and Co-WIN experiences; and third, treatment of governance, accountability, and redress mechanisms as a standalone pillar rather than a subsidiary consideration.

**Fig. 2:**
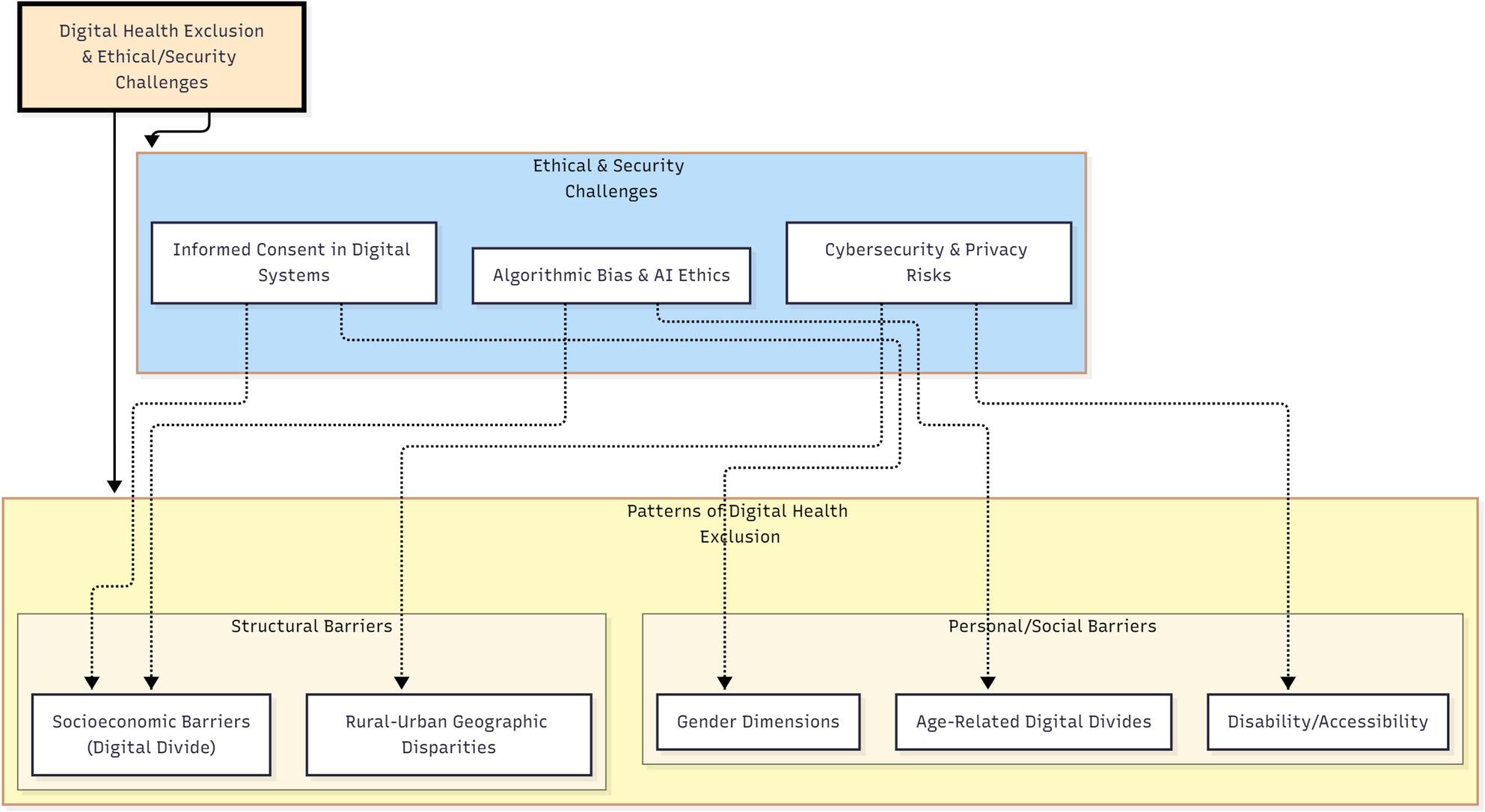
Integrated framework of digital health exclusion and ethical/security challenges. This conceptual model illustrates the multi-dimensional nature of digital health exclusion, organized into structural barriers (socioeconomic disparities and rural-urban geographic divides, shown in yellow) and personal/social barriers (gender dimensions, age-related digital divides, and disability/accessibility gaps, shown in yellow). These exclusion patterns interact with three critical ethical and security challenges (shown in the blue panel): (1) informed consent in digital systems, (2) algorithmic bias and AI ethics, and (3) cybersecurity and privacy risks. The dotted lines represent dynamic interactions between exclusion patterns and ethical challenges, emphasizing that exclusion is not merely about access but also about how individuals are represented, protected, and empowered within digital health systems. Understanding these intersections is essential for developing equity-focused digital health policies and implementation strategies that address root causes rather than symptoms of exclusion

### Framework components

#### Multi-channel service delivery

##### Principle

Digital health should expand rather than replace service delivery options.

**Implementation strategies**:

- Inclusive Training: Invest in digital literacy programs for patients and providers, tailored to diverse needs and contexts.
- Digital and Non-Digital Options: Maintain robust non-digital service channels (in-person, telephone, assisted digital) alongside digital offerings.
- Hybrid Models: Design services that allow flexible switching between modalities based on user preference and context.
- Assisted Services: Provide trained navigators or community health workers to support individuals needing assistance with digital systems.
- Stigma-Free Access: Ensure that non-digital options are not degraded, delayed, or stigmatized relative to digital services.

#### Digital literacy and capacity building

##### Principle

Effective digital health engagement requires investment in human capacity, not just technology.

**Implementation strategies**:

- Community Training Programs: Develop culturally appropriate, multilingual digital literacy programs accessible to diverse populations.
- Population Literacy Assessment: Conduct baseline assessments of digital literacy across target populations to inform program design.
- Provider Skills Development: Provide comprehensive training for healthcare workers in system use, workflow integration, and troubleshooting.
- User-Friendly Design: Prioritize intuitive interfaces, clear instructions, and simplified navigation that minimize literacy requirements.
- Ongoing Support: Establish accessible technical support channels (helplines, in-person assistance, peer support networks).

#### Participatory design and co-design

##### Principle

Those most affected by digital health systems must have meaningful voice in their design and governance.

**Implementation strategies**:

- Patient Advisory Boards: Establish standing advisory groups including representatives from marginalized communities.
- Engage End-Users: Involve patients and community members throughout the design process, from needs assessment through testing and refinement.
- Community Health Workers: Engage frontline workers who understand community needs and can bridge between systems and users.
- Community Consultation: Conduct regular consultations with community organizations, advocacy groups, and civil society.
- Power Sharing: Ensure participatory processes include genuine decision-making authority, not just consultation.

#### Governance and accountability

##### Principle

Digital health systems require transparent governance with clear accountability for equity outcomes.

**Implementation strategies**:

- Community Governance: Include community representatives in governance structures for digital health programs.
- Privacy and Security: Implement robust data protection policies with privacy-by-design and security-by-design architectures.
- Transparent Algorithms: Require disclosure of AI system training data, validation results, and performance across population subgroups.
- Redress Mechanisms: Establish accessible processes for users to report problems, seek redress for harms, and influence system improvement.
- Oversight: Create independent oversight bodies with authority to audit systems, investigate complaints, and mandate corrective action.

#### Equity monitoring and evaluation

##### Principle

What gets measured gets managed, equity must be continuously monitored and evaluated.

**Implementation strategies**:

- Differential Outcomes: Track health outcomes disaggregated by socioeconomic status, geography, gender, age, disability, and other equity-relevant dimensions.
- Disaggregated Metrics: Collect and analyze usage data disaggregated by population subgroups to identify exclusion patterns.
- Monitor Exclusion: Systematically track who is not using services and investigate barriers.
- User Experience Research: Conduct regular qualitative research with diverse users to understand experiences, challenges, and needs.
- Continuous Adaptation: Use monitoring data to iteratively refine systems, address barriers, and improve equity outcomes.

### Stakeholder-specific guidance

**Fig. 3:**
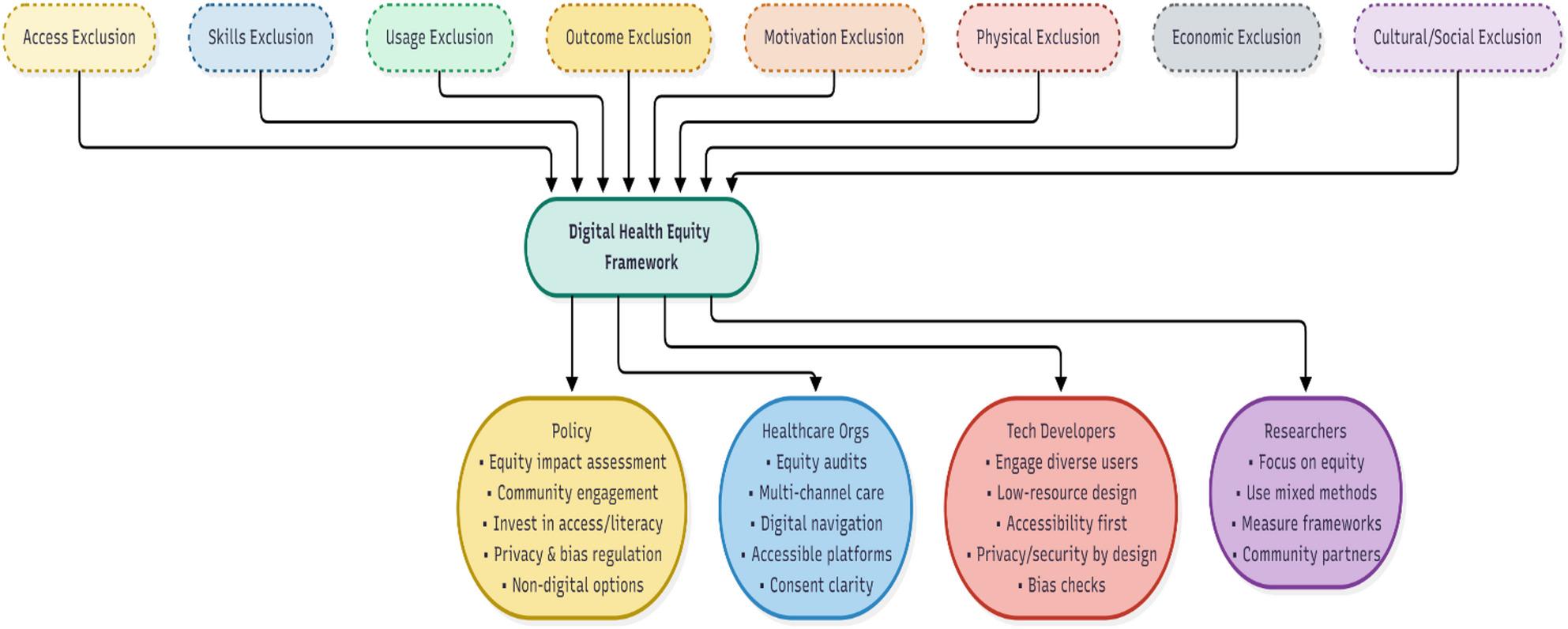
Stakeholder-specific applications of the digital health equity framework. This figure illustrates how the integrated equity framework translates into differentiated guidance for four key stakeholder groups. At the center, the Digital Health Equity Framework serves as the common foundation, addressing eight types of exclusion: Access Exclusion, Skills Exclusion, Usage Exclusion, Outcome Exclusion, Motivation Exclusion, Physical Exclusion, Economic Exclusion, and Cultural/Social Exclusion. From this central framework, specific action domains radiate outward to each stakeholder group: (1) Policy stakeholders (shown in yellow) focus on equity impact assessment, community engagement, investment in access and literacy infrastructure, privacy and bias regulation, and ensuring non-digital options remain available; (2) Healthcare Organizations (shown in blue) prioritize equity audits, multi-channel care delivery, digital navigation support, accessible platforms, and consent clarity; (3) Tech Developers (shown in red) emphasize engaging diverse users in design, low-resource design principles, accessibility-first development, privacy and security by design, and systematic bias checks; and (4) Researchers (shown in purple) focus on equity-centered research questions, mixed-methods approaches, framework measurement and validation, community partnership, and implementation science. This stakeholder-specific translation recognizes that achieving digital health equity requires coordinated action across multiple sectors, each contributing their unique capabilities and responsibilities. The framework provides a common language and shared goals while respecting the distinct roles and leverage points of different actors in the digital health ecosystem

The framework translates into differentiated guidance for key stakeholder groups (Fig. 3):

#### Policy and government

##### Equity impact assessment

Require prospective equity impact assessments for all digital health initiatives, examining potential effects on vulnerable populations.

##### Community engagement

Mandate meaningful community participation in digital health planning and governance.

##### Invest in access and literacy

Allocate resources for infrastructure, connectivity, devices, and digital literacy programs targeting underserved populations.

##### Privacy and bias regulation

Establish strong data protection laws and algorithmic accountability requirements with effective enforcement.

##### Non-digital options

Require that digital health programs maintain robust non-digital service alternatives.

#### Healthcare organizations

##### Equity audits

Conduct regular audits of digital health service utilization and outcomes across population subgroups.

##### Multi-channel care

Implement integrated service delivery that allows patients to access care through their preferred modality.

##### Digital navigation

Employ trained patient navigators to support individuals needing assistance with digital systems.

##### Accessible platforms

Ensure digital health platforms meet accessibility standards for persons with disabilities.

##### Consent clarity

Implement clear, understandable consent processes that respect patient autonomy and privacy.

#### Technology developers

##### Engage diverse users

Involve representatives from marginalized communities throughout the design process.

##### Low-resource design

Design systems that function in low-resource contexts (limited connectivity, older devices, low digital literacy).

##### Accessibility first

Build accessibility features from the outset rather than as afterthoughts.

##### Privacy and security by design

Implement robust security architectures and privacy-preserving technologies.

##### Bias checks

Conduct rigorous testing for algorithmic bias across diverse populations and contexts.

#### Researchers

##### Focus on equity

Prioritize research questions examining equity dimensions of digital health.

##### Use mixed methods

Employ both quantitative and qualitative approaches to understand exclusion mechanisms and user experiences.

##### Measure frameworks

Develop and validate metrics for assessing digital health equity across multiple dimensions.

##### Community partners

Partner with community organizations and affected populations in research design and interpretation.

##### Implementation science

Study not just whether digital health works but how to implement it equitably across diverse contexts.

## Discussion

### Principal findings

This narrative review synthesized evidence from 22 peer-reviewed and policy sources examining digital health equity across diverse contexts, with particular attention to implementation experiences from India and comparable LMIC settings. Four principal findings emerge:

**First**, digital health interventions frequently mirror and amplify existing health inequities rather than reducing them. Patterns of exclusion operate across multiple intersecting dimensions, socioeconomic status, geography, gender, age, disability, and digital literacy, creating cumulative disadvantage for marginalized populations. The digital divide is not merely about access to devices and connectivity but encompasses deeper structural barriers related to literacy, language, accessibility, and power.

**Second**, ethical challenges in digital health, including inadequate consent processes, persistent algorithmic bias, and escalating cybersecurity risks, disproportionately affect vulnerable populations who have the least power to protect their interests and the most to lose from privacy breaches or discriminatory algorithms. Current ethical frameworks and regulatory structures are inadequate for the complexity and scale of contemporary digital health systems.

**Third**, implementation experiences reveal critical gaps between policy aspirations and operational reality. Fragmented governance, insufficient infrastructure investment, limited provider capacity, weak community engagement, and inadequate equity monitoring undermine even well-intentioned digital health initiatives. “Digital-by-default” approaches that eliminate non-digital options systematically exclude populations facing access or literacy barriers.

**Fourth**, addressing these challenges requires integrated attention to exclusion patterns, ethical safeguards, and implementation strategies. Existing frameworks have tended to address these dimensions in isolation; our proposed integrated framework explicitly links them, providing actionable guidance for equity-focused digital health implementation.

### Implications for policy and practice

#### Equity must be designed, not assumed

Digital health will not inherently advance equity; rather, equity must be intentionally designed into systems from the outset [2, 6]. This requires:

- Prospective equity impact assessments that examine potential effects on vulnerable populations before implementation.
- Participatory design processes that center the voices and needs of marginalized communities.
- Multi-channel service delivery that expands rather than replaces access options.
- Routine equity monitoring with disaggregated data collection and analysis.
- Accountability mechanisms that enable redress when systems cause harm or exclusion.

#### Infrastructure and capacity investment

Sustainable digital health requires foundational investments that extend beyond software development to include:

- Telecommunications infrastructure ensuring reliable connectivity in underserved areas.
- Device access programs providing smartphones or tablets to low-income populations.
- Digital literacy programs tailored to diverse communities and age groups.
- Provider training and support enabling effective system use and workflow integration.
- Technical support infrastructure providing ongoing assistance to users and providers.

#### Governance and regulation

Effective governance requires:

- Data protection laws with meaningful enforcement and penalties for violations.
- Algorithmic accountability requirements including transparency, bias testing, and impact assessments.
- Independent oversight bodies with authority to audit systems and mandate corrective action.
- Community representation in governance structures for digital health programs.
- Redress mechanisms enabling users to report problems and seek remedies.

#### Preserving non-digital options

Digital transformation should not eliminate non-digital service pathways. Maintaining robust alternatives is essential for equity and requires:

- Sustained investment in non-digital channels rather than viewing digital as a cost-saving replacement.
- Parity of service quality ensuring non-digital options are not degraded or delayed.
- Assisted digital services providing support for individuals who need help navigating digital systems.
- Flexible modality switching allowing individuals to move between digital and non-digital options based on preference and context.

### Implications for research

Critical research gaps include:

#### Measurement and metrics

Development and validation of comprehensive metrics for assessing digital health equity across multiple dimensions, including access, usage, outcomes, and experience.

#### Implementation science

Research examining how to implement digital health equitably across diverse contexts, including identification of effective strategies for community engagement, provider capacity building, and equity monitoring.

#### Intersectionality

Studies examining how multiple dimensions of marginalization (e.g., low income, rural location, female gender, older age) interact to shape digital health access and outcomes.

#### Long-term outcomes

Longitudinal research assessing whether digital health interventions narrow or widen health equity gaps over time, with attention to unintended consequences.

#### Governance models

Comparative research on governance structures for digital health, examining effectiveness of different approaches to community participation, algorithmic accountability, and data protection.

#### Economic evaluation

Cost-effectiveness analyses that incorporate equity considerations, examining not just average effects but differential impacts across population subgroups.

### Strengths and limitations

This review’s strengths include its comprehensive scope across exclusion patterns, ethical challenges, and implementation experiences; explicit focus on equity dimensions often overlooked in digital health literature; grounding in real-world implementation experiences from diverse contexts; and development of an integrated framework linking analysis to actionable guidance.

Limitations include the narrative synthesis approach, which does not permit meta-analysis or formal quality assessment; potential publication bias toward positive findings or well-resourced settings; rapid evolution of the digital health field, meaning some evidence may quickly become outdated; and limited availability of rigorous implementation research from many LMIC contexts. Additionally, while we prioritized recent literature (2015–2025), some relevant earlier work may have been excluded. The reference list is intentionally selective, representing the 22 sources most directly informative to the synthesis domains and framework development rather than an exhaustive census of the literature. Peer-reviewed primary outcome evaluation studies specifically focused on ABDM remain limited and emerging at the time of this review, and the NHA Annual Report used as a primary source [23] carries the inherent limitations of government-produced programme documentation.

### Future directions

Digital health is evolving rapidly, with emerging technologies and implementation models creating both new opportunities and new risks for equity. Priority areas for future attention include:

#### Artificial Intelligence

As AI becomes more deeply embedded in clinical decision-making, ensuring algorithmic fairness, transparency, and accountability is increasingly urgent.

#### Data sovereignty

Debates about data ownership, control, and governance, particularly for Indigenous populations and other marginalized communities, require careful attention to power dynamics and historical injustices.

#### Commercial determinants

The growing role of commercial technology companies in healthcare raises questions about whose interests are prioritized, how data is used, and whether profit motives align with health equity goals.

#### Climate and digital health

Understanding how climate change intersects with digital health equity, including infrastructure vulnerabilities, displacement effects, and differential climate impacts on marginalized populations.

#### Pandemic preparedness

Applying lessons from COVID-19 digital health responses to build more equitable systems for future health emergencies.

## Conclusion

Digital health holds genuine promise for advancing health equity, but only if equity is intentionally designed, systematically governed, and continuously monitored. The evidence synthesized in this review demonstrates that digital transformation can either close or widen health equity gaps depending on how it is implemented.

Our integrated Digital Health Equity Framework provides a roadmap for more inclusive digital health implementation, linking patterns of exclusion with ethical safeguards and implementation strategies. By applying this framework, policymakers, health system leaders, technology developers, and researchers can work toward digital health systems that genuinely serve all populations, ultimately advancing universal health coverage through inclusive digital transformation.

The path forward requires sustained commitment to equity as a core design principle, meaningful participation of marginalized communities in digital health governance, preservation of non-digital service options, robust data protection and algorithmic accountability, and continuous monitoring and adaptation based on equity outcomes. Digital health will not automatically democratize healthcare, but with intentional effort, it can become a powerful tool for advancing health equity rather than perpetuating exclusion.

## Supplementary Information

Below is the link to the electronic supplementary material.

Supplementary Material 1

## Data Availability

No datasets were generated or analysed during the current study.

## References

[1] Xavier PB, Silva I, de Figueiredo S, de Araújo RC, de Silva AJ. AJB da, Uchôa SA da C. Impact of digital health on the quality of primary care for people with chronic noncommunicable diseases: a scoping review protocol. PLOS ONE. 2025;20(1):e0316278. 10.1371/journal.pone.0316278. 10.1371/journal.pone.0316278 PMC11844851 39982873

[2] Richardson S, Lawrence K, Schoenthaler AM, Mann D. A framework for digital health equity. NPJ Digit Med. 2022;5(1):119. 10.1038/s41746-022-00663-0. 35982146 10.1038/s41746-022-00663-0 PMC9387425

[3] Hollimon LA, Taylor KV, Fiegenbaum R, Carrasco M, Garchitorena Gomez L, Chung D, et al. Redefining and solving the digital divide and exclusion to improve healthcare: going beyond access to include availability, adequacy, acceptability, and affordability. Front Digit Health. 2025;7:1508686. 10.3389/fdgth.2025.1508686. 40330871 10.3389/fdgth.2025.1508686 PMC12052546

[4] Sylla B, Ismaila O, Diallo G. 25 Years of Digital Health Toward Universal Health Coverage in Low- and Middle-Income Countries: Rapid Systematic Review. J Med Internet Res. 2025;27:e59042. 10.2196/59042. 40440696 10.2196/59042 PMC12163355

[5] Hasanzadeh F, Josephson CB, Waters G, Adedinsewo D, Azizi Z, White JA. Bias recognition and mitigation strategies in artificial intelligence healthcare applications. NPJ Digit Med. 2025;8(1):154. 10.1038/s41746-025-01434-9. 40069303 10.1038/s41746-025-01503-7 PMC11897215

[6] Hatef E, Hudson Scholle S, Buckley B, Weiner JP, Austin JM. Development of an evidence- and consensus-based Digital Healthcare Equity Framework. JAMIA Open. 2024;7(4):ooae136. 10.1093/jamiaopen/ooae136. 39553827 10.1093/jamiaopen/ooae136 PMC11565864

[7] Groom LL, Schoenthaler AM, Mann DM, Brody AA. Construction of the Digital Health Equity-Focused Implementation Research Conceptual Model – Bridging the Divide Between Equity-focused Digital Health and Implementation Research. PLOS Digit Health. 2024;3(5):e0000509. 10.1371/journal.pdig.0000509. 38776354 10.1371/journal.pdig.0000509 PMC11111026

[8] Kim KK, Backonja U. Digital health equity frameworks and key concepts: a scoping review. J Am Med Inf Assoc. 2025;32(4):932–44. 10.1093/jamia/ocae308. 10.1093/jamia/ocaf017 PMC12012343 39936843

[9] Kepper MM, Fowler LA, Kusters IS, Davis JW, Baqer M, Sagui-Henson S, et al. Expanding a Behavioral View on Digital Health Access: Drivers and Strategies to Promote Equity. J Med Internet Res. 2024;26:e51355. 10.2196/51355. 39088246 10.2196/51355 PMC11327633

[10] World Health Organization. Global strategy on digital health 2020–2025. Geneva: WHO. 2021. Available from: https://www.who.int/docs/default-source/documents/gs4dhdaa2a9f352b0445bafbc79ca799dce4d.pdf.

[11] Buslón N, Cortés A, Catuara-Solarz S, Cirillo D, Rementeria MJ. Raising awareness of sex and gender bias in artificial intelligence and health. Front Glob Womens Health. 2023;4:970312. 10.3389/fgwh.2023.970312. 37746321 10.3389/fgwh.2023.970312 PMC10512182

[12] Richards N, Hartzog W. The pathologies of digital consent. Wash Univ Law Rev. 2021;96(6):1461–503. Available from: https://openscholarship.wustl.edu/law_lawreview/vol96/iss6/3/.

[13] Nandra R, Brockie AF, Hussain F. A review of informed consent and how it has evolved to protect vulnerable participants in emergency care research. EFORT Open Rev. 2020;5(2):73–9. 10.1302/2058-5241.5.190026. 32175093 10.1302/2058-5241.5.180051 PMC7047905

[14] Shivayogi P. Vulnerable population and methods for their safeguard. Perspect Clin Res. 2013;4(1):53–7. 10.4103/2229-3485.106389. 23533983 10.4103/2229-3485.106389 PMC3601707

[15] Kaye J, Whitley EA, Lund D, Morrison M, Teare H, Melham K. Dynamic consent: a patient interface for twenty-first century research networks. Eur J Hum Genet. 2015;23(2):141–6. 10.1038/ejhg.2014.71. 24801761 10.1038/ejhg.2014.71 PMC4130658

[16] Balestra M, Shaer O, Okerlund J, Westendorf L, Ball M, Nov O. Social Annotation Valence: The Impact on Online Informed Consent Beliefs and Behavior. J Med Internet Res. 2016;18(8):e197. 10.2196/jmir.5672. 27439320 10.2196/jmir.5662 PMC4972991

[17] Haider SA, Borna S, Gomez-Cabello CA, Pressman SM, Haider CR, Forte AJ. The Algorithmic Divide: A Systematic Review on AI-Driven Racial Disparities in Healthcare. J Racial Ethn Health Disparities. 2024. 10.1007/s40615-024-02177-4. 39695057 10.1007/s40615-024-02237-0

[18] Cross JL, Choma MA, Onofrey JA. Bias in medical AI: Implications for clinical decision-making. PLOS Digit Health. 2024;3(11):e0000651. 10.1371/journal.pdig.0000651. 39509461 10.1371/journal.pdig.0000651 PMC11542778

[19] Luna R, Rhine E, Myhra M, Sullivan R, Kruse CS. Cyber threats to health information systems: A systematic review. Technol Health Care. 2016;24(1):1–9. 10.3233/THC-151102. 26578272 10.3233/THC-151102

[20] Ewoh P, Vartiainen T. Vulnerability to Cyberattacks and Sociotechnical Solutions for Health Care Systems: Systematic Review. J Med Internet Res. 2024;26:e46904. 10.2196/46904. 38820579 10.2196/46904 PMC11179043

[21] Wasserman L, Wasserman Y. Hospital cybersecurity risks and gaps: Review (for the non-cyber professional). Front Digit Health. 2022;4:862221. 10.3389/fdgth.2022.862221. 36033634 10.3389/fdgth.2022.862221 PMC9403058

[22] Seh AH, Zarour M, Alenezi M, Sarkar AK, Agrawal A, Kumar R, et al. Healthcare Data Breaches: Insights and Implications. Healthc (Basel). 2020;8(2):133. 10.3390/healthcare8020133. 10.3390/healthcare8020133 PMC7349636 32414183

[23] National Health Authority, Government of India. Ayushman Bharat Digital Mission: annual report 2022–23. New Delhi: Ministry of Health and Family Welfare. 2023. Available from: https://abdm.gov.in.

