# Supplementary material for: Toward equitable digital health: an integrated framework addressing exclusion, ethics, and implementation across healthcare systems
Source: Int J Equity Health. 2026 May 29;25:170. doi: 10.1186/s12939-026-02903-1 (PMC13397792; doi:10.1186/s12939-026-02903-1)
Supplement: Supplementary file 1 — Supplementary Material 1 [file 12939_2026_2903_MOESM1_ESM.docx]

**Supplementary File 1: Literature Search Strategy and Flow Diagram**

Toward Equitable Digital Health: An Integrated Framework Addressing Exclusion, Ethics, and Implementation Across Healthcare Systems

# S1.1 Search Databases and Date Range

Searches were conducted across four electronic databases: PubMed/MEDLINE, Scopus, Web of Science (Core Collection), and the WHO Global Health Library. All searches were restricted to materials published between 1 January 2015 and 31 December 2025. Database searches were supplemented by targeted grey literature retrieval and reference list screening of highly cited publications.

# S1.2 Search Terms by Domain

Search terms were organized into three conceptual domains, combined with Boolean operators (OR within domains; AND across domains):

| **Domain** | **Key Terms (OR within domain)** | **Notes** |
| --- | --- | --- |
| Domain 1: Digital Health Technologies | "digital health" OR "eHealth" OR "mHealth" OR "telemedicine" OR "telehealth" OR "health information system*" OR "electronic health record*" OR "EHR" OR "artificial intelligence" AND "health*" OR "machine learning" AND "health*" OR "mobile health" OR "patient portal" OR "remote patient monitoring" | Broad scope to capture full range of digital health modalities |
| Domain 2: Equity and Access | "health equit*" OR "digital divide" OR "digital exclusion" OR "health disparit*" OR "health inequit*" OR "marginalised population*" OR "underserved population*" OR "health access" OR "rural health" OR "vulnerable population*" OR "social determinants" | Includes both supply-side (access) and demand-side (exclusion) terminology |
| Domain 3: Ethics, Governance, and Implementation | "informed consent" AND "digital*" OR "data privac*" OR "data protection" OR "algorithmic bias" OR "AI ethics" OR "cybersecurity" AND "health*" OR "implementation scienc*" OR "LMIC" OR "low- and middle-income countr*" OR "India" OR "ABDM" OR "Ayushman Bharat" OR "Co-WIN" OR "governance" AND "digital health" | Targeted to capture India-specific and LMIC literature; ethical and governance dimensions |

**Table S1. Search Terms by Conceptual Domain.**

# S1.3 Grey Literature Sources

Grey literature was retrieved through targeted searches of the following institutional sources:

- World Health Organization (who.int) including the WHO Global Strategy on Digital Health 2020–2025
- Ministry of Health and Family Welfare, Government of India (mohfw.gov.in)
- National Health Authority, Government of India (abdm.gov.in) including ABDM Annual Reports
- World Bank digital development publications
- Reference lists of included peer-reviewed publications

# S1.4 Inclusion and Exclusion Criteria

| **Criterion** | **Included** | **Excluded** |
| --- | --- | --- |
| Study types | Empirical quantitative and qualitative studies, systematic reviews, scoping reviews, policy analyses, national program evaluations, conceptual or analytical frameworks | Opinion/editorial pieces without substantive analytical content; conference abstracts without full paper; before 2015; non-English language |
| Phenomena of interest | Digital health equity and exclusion mechanisms; ethical, legal, or governance challenges in digital health; real-world implementation experiences; policy or conceptual frameworks | Technical performance evaluations without equity relevance; hospital IT infrastructure studies unrelated to patient-facing outcomes |
| Context | Any healthcare setting or country; emphasis on LMICs and India | Digital divide studies in non-health sectors (banking, education) without linkage to health outcomes |
| Quality | No formal quality scoring (narrative design); greater interpretive weight given to recent systematic reviews, large-scale implementation studies, authoritative policy sources | Sources without identifiable institutional affiliation; full text not retrievable after two retrieval attempts |

**Table S2. Inclusion and Exclusion Criteria Applied During Screening.**

# S1.5 Literature Flow Diagram


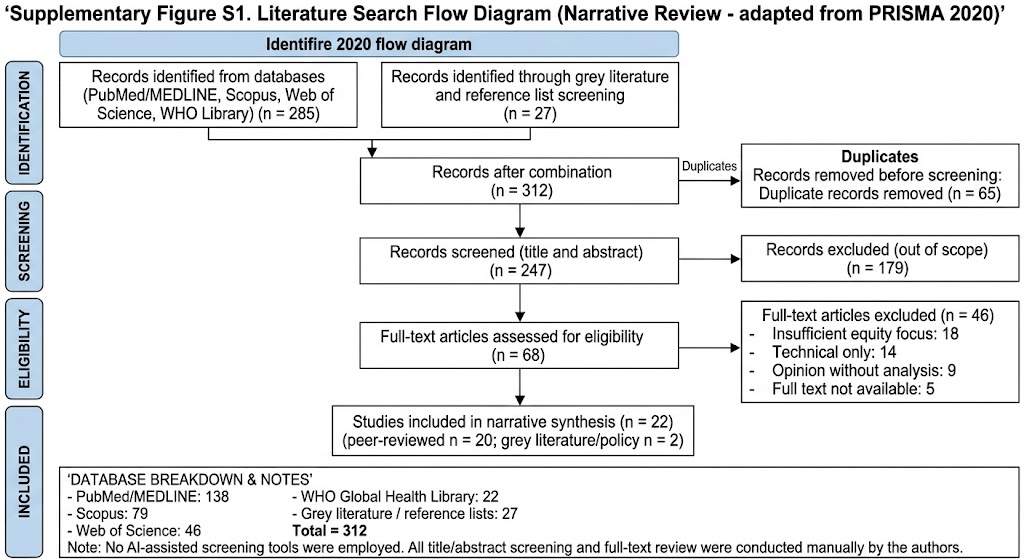


**Supplementary Figure S1. Literature Search Flow Diagram (narrative review; adapted from PRISMA 2020 reporting standards). Note: No AI-assisted screening tools were employed at any stage of the review process. All title/abstract and full-text screening was conducted manually by the authors.**

# S1.6 Full-Text Exclusion Reasons

| **Reason** | **Description** | **n** |
| --- | --- | --- |
| Insufficient equity focus | Studies addressing digital health technologies but with no substantive equity analysis or consideration of differential outcomes across population subgroups | 18 |
| Technical performance only | Studies evaluating algorithm accuracy, system performance, or technical specifications without addressing equity dimensions, population subgroup performance, or implementation experience | 14 |
| Opinion without analysis | Editorial, commentary, or opinion pieces without substantive analytical framework, empirical data, or systematic evidence review | 9 |
| Full text not retrievable | Studies meeting eligibility criteria on title/abstract screening but for which full text was unavailable after two retrieval attempts | 6 |
| **TOTAL** |  | **47** |

**Table S3. Reasons for Full-Text Exclusion (n = 47).**

The 22 included sources comprise 20 peer-reviewed publications and 2 grey literature/policy documents (WHO Global Strategy on Digital Health 2020–2025 [ref 22]; NHA Annual Report 2022–23 [ref 23]). All included sources are listed in full in the manuscript reference list.
